# Efficacy and safety of ^177^Lu-DOTATATE targeted therapy in advanced/metastatic pulmonary neuroendocrine tumors: A systematic review and meta-analysis

**DOI:** 10.3389/fonc.2022.993182

**Published:** 2022-11-24

**Authors:** Jiao Ma, Xin Hu, Lanying Li, Zijuan Rao, Chunyin Zhang

**Affiliations:** ^1^ Department of Nuclear Medicine, The Affiliated Hospital of Southwest Medical University, Luzhou, Sichuan, China; ^2^ Department of Orthopedics, Second People’s Hospital of Yibin City, Yibin, Sichuan, China; ^3^ Nuclear Medicine and Molecular Imaging Key Laboratory of Sichuan Province, Luzhou, Sichuan, China; ^4^ Academician (expert) Workstation of Sichuan Province, Luzhou, Sichuan, China

**Keywords:** ^177^Lu-DOTATATE, radionuclide therapy, meta-analysis, lung, neuroendocrine tumors

## Abstract

**Objective:**

To perform a meta-analysis of the efficacy and safety about ^177^Lu-DOTATATE therapy for advanced/metastatic pNETs based on the current clinical evidence.

**Methods:**

This systematic review follows the PRISMA guideline. Search PubMed, Medline, EMBASE and CNKI, VIP, Wanfang databases, from establishment to June 2022, on the study of ^177^Lu-DOTATATE for advanced/metastatic pNETs, the primary endpoint was to evaluate the treatment effect through DRRs and DCRs. Secondary endpoint included assessment of OS, PFS, and treatment-related adverse events across all studies. Two researchers conducted literature screening, data extraction and quality evaluation according to the inclusion and exclusion criteria. Meta-analysis was performed using stata16.0 software, and the data were merged and displayed using forest graphs.

**Results:**

A total of 5 studies, 174 patients, on ^177^Lu-DOTATATE for advanced/metastatic pNETs were included. The pools of DRRs and DCRs were 24% (95% CI: 15%~32%) and 77% (95% CI: 62%~92%), respectively. The pool of OS was 48.78 months (95% CI: 41~56.57 months) and the pool of PFS was 21.59 months (95% CI: 17.65~25.53 months). In all studies, the most common side effect of treatment was hematological toxicity. In 174 patients, hematological toxicity of grade III accounted for 4.0% (7/174), and only 4.0% (7/174) and 1.0% (2/174) of patients had mild nephrotoxicity and hepatotoxicity. Gastrointestinal adverse reactions in 3% (6/174), nausea in 2% (3/174), superior vena cava occlusion in 0.5% (1/174).

**Conclusion:**

^177^Lu-DOTATATE is effective and safe for advanced/metastatic pNETs, which can delay the progression of the disease, may improve patients’ survival, and has low treatment-related toxicity and high safety. However, its efficacy and safety need to be further evaluated in high-quality, multicenter randomized controlled trials in the future.

**Systematic review registration:**

https://www.crd.york.ac.uk/PROSPERO/, identifier CRD42022344436.

## Introduction

Neuroendocrine tumors(NETs) are a family of malignancies of diverse origin, including the lung, gastrointestinal tract, and pancreas. Among them, pulmonary neuroendocrine tumors (pNETs) are a type of epithelial tumor originating from the neuroendocrine cells of the lung, accounting for about 1-2% of all lung cancers, accounting for 20-25% of all NETs. WHO divides it into 4 subtypes: typical carcinoid (TC) (2%), atypical carcinoid (AC) (<1%), large cell neuroendocrine carcinoma (LCNEC) (3%) and small cell lung cancer (SCLC) (20%) (1). In recent years, with the increase of awareness of pNETs, the advancement of imaging technology and the early screening of lung cancer in smokers, the incidence and prevalence of pNETs have increased linearly (2). TC and AC are called pulmonary carcinoid tumors. Compared with poorly differentiated NETs, TC and AC patients have relatively lower disease malignancy and invasiveness, and have better prognosis and survival. Radical surgery is currently the gold standard of treatment. However, some patients still have recurrence or distant metastasis after radical surgery (3). For patients in the advanced/metastatic stage, treatment aims to prolong overall survival (OS) and maintain quality of life by controlling hormone production and preventing disease progression. There is still a need to continuously explore more effective, individualized and precise treatments. Compared with gastrointestinal neuroendocrine tumors, lung carcinoid patients rarely show carcinoid syndrome, with an incidence of only 1% to 5% (4), and the appearance of symptoms may indicate tumor metastasis. For patients with hormone-related symptoms, somatostatin analogues (SSAs) are recommended as first-line treatment options by guidelines such as the European neuroendocrine Tumor Society (ENETS), especially for patients with low proliferative index (Ki-67<10%), SSTR positive, slow progression. 5% to 10% of patients have partial response (PR), 30% to 50% of patients have stable disease (SD), and 40% to 60% of patients have symptomatic improvement. However, the application in non-functional tumors is still controversial (4). The overall response rate of chemotherapy drugs such as doxorubicin, 5-fluorouracil, dacarbazine, and cisplatin in the treatment of advanced lung carcinoids is 20% to 30%, but there are many adverse reactions. Temozolomide is the most widely studied drug in pNETs with high safety and is recommended as a drug, often combined with other antitumor drugs for metastatic pNETs. Currently, the Food and Drug Administration (FDA) has only approved everolimus for patients with advanced pulmonary typical/atypical carcinoid tumors, which can improve progression-free survival (PFS) (5). Peptide receptor radionuclide therapy (PRRT) is a promising treatment for patients with advanced or inoperable somatostatin receptor-positive tumors. The FDA has approved ^177^Lu-DOTATATE mediated PRRT for gastroenteropancreatic neuroendocrine tumors (6). In the latest NETTER-1 trial on midgut neuroendocrine tumors, ^177^Lu-Dotatate has showed improved OS in patients compared with high-dose long-acting octreotide alone (7). However, the trial did not include pNETs. Only a few studies have evaluated the efficacy of ^177^Lu-DOTATATE in pNETs, and there is no systematic review or meta-analysis on the efficacy and safety of ^177^Lu-DOTATATE for pNETs in the published literature. In this study, we conducted a meta-analysis of the published clinical studies of ^177^Lu-DOTATATE for advanced/metastatic pNETs, in order to provide a reference for clinical selection of more effective and individualized treatment options for patients at this stage and to provide some evidence-based medical evidence for the efficacy and safety of ^177^Lu-DOTATATE in the treatment of metastatic pNETs.

## Materials and methods

This systematic review followed the Preferred Reporting Items for Systematic Reviews and Meta-Analyses (PRISMA) guideline

### Search strategy

Search in PubMed, Medline, EMBASE and CNKI, VIP, Wanfang databases, from establishment to June 2022, on the study of ^177^Lu-DOTATATE for advanced/metastatic pNETs. The search used a combination of subject words and free words. Search terms: {(“neuroendocrine tumor*”[Mesh] OR “neuroendocrine tumour*”OR”neuroendocrine neoplasm*”OR “neuroendocrine cancer*” OR “neuroendocrine carcinoma*”AND (Lutetium-177 [Mesh] OR ^177^Lu OR 177Lutetium OR Lu-177) AND (Lung [MeSH]) OR Pulmonary)}.Search for all clinical studies on ^177^Lu-labeled radiopharmaceuticals in the treatment of pNETs. These articles were evaluated for response to treatment using the Response Evaluation Criteria in Solid Tumours (RECIST) 1.1 or Southwest Oncology Group (SWOG) criteria, and appropriate data were included for analysis. Full text will be searched when the article meets the research criteria. If there were duplications (patient data from the same trial or institution), only the most complete, current, and relevant study was selected. Two researchers independently searched the literature and extracted data. If there was a disagreement, discussed and resolved with a third party. A total of 5 articles were included, and the methodological quality of the literatures were assessed using the methodological index for non-randomized studies (MINORS).

### Study selection

#### Inclusion criteria

① No less than 10 patients with advanced/metastatic pNETs confirmed by biopsy, laboratory examination and imaging examination; ^68^Ga-DOTATATE PET/CT showed high affinity for SSR receptor imaging in lesions (^68^Ga-DOTATATE uptake was higher than liver activity, Krenning score 3-4). If data were from the same study group, the study with the highest number of patients was included. ② Complete at least 1 cycle of ^177^Lu-DOTATATE treatment; ③ Studies were grouped according to the criteria used for response assessment in the text. The main study indicators included disease response rates (DRRs) and disease control rates (DCRs). ④ Retrospective/prospective studies.

#### Exclusion criteria

①Patients with severe hypoalbuminemia, leukopenia, thrombocytopenia, liver and kidney failure; ②^68^Ga-DOTATATE PET/CT showed low affinity for SSR receptor imaging in the lesion (^68^Ga-DOTATATE uptake equal to or lower than liver activity, Krenning score 0-2); ③The study did not provide infusion amino acids for kidney protection; ④Duplicate published studies, conferences, meta-analyses, reviews, case reports, brief communications, abstracts, letters to the editor.

### Data extraction

The basic characteristics of the studies include the first author, publication time, demographic characteristics such as the number and age of the study population, study type, previous treatments, metastases, treatment response evaluation criteria, radiopharmaceutical treatment regimens and doses, etc. Outcomes in the studies included DRRs, DCRs, PFS, OS, and toxicity. DRRs is defined as the percentage of complete response (CR) + partial response (PR); DCRs is defined as the percentage of complete response (CR) + partial response (PR) + stable disease (SD). PFS is defined as the time from the first dose of ^177^Lu-DOTATATE to the first evidence of progression or death or the end of the study period; OS is defined as the time from the first dose of ^177^Lu-DOTATATE to death for any cause. Toxicity is defined according to the Common Terminology Criteria for Adverse Events (CTCAE3.0-5.0) (8–10).

### Data analysis

Meta-analysis is performed using STATA16.0. The primary outcome are DRRs and DCRs as assessed by RECIST1.1 or SWOG. Secondary outcome include OS, PFS, and treatment-related toxicity. Draw a forest plot for analysis. I^2^ statistic is used to test for heterogeneity. If there is no significant heterogeneity among studies (I^2^ ≤ 50%, P<0.10), a fixed effect model is used to combine data. If there is significant heterogeneity among studies (I^2^>50%, P≥0.10), a random effect model is used to combine data. Funnel plot and Egger test are used to evaluate the publication bias of imaging response after ^177^Lu-DOTATATE treatment, and P ≤ 0.05 was considered statistically significant.

## Result

### Literature search

**Figure 1:**
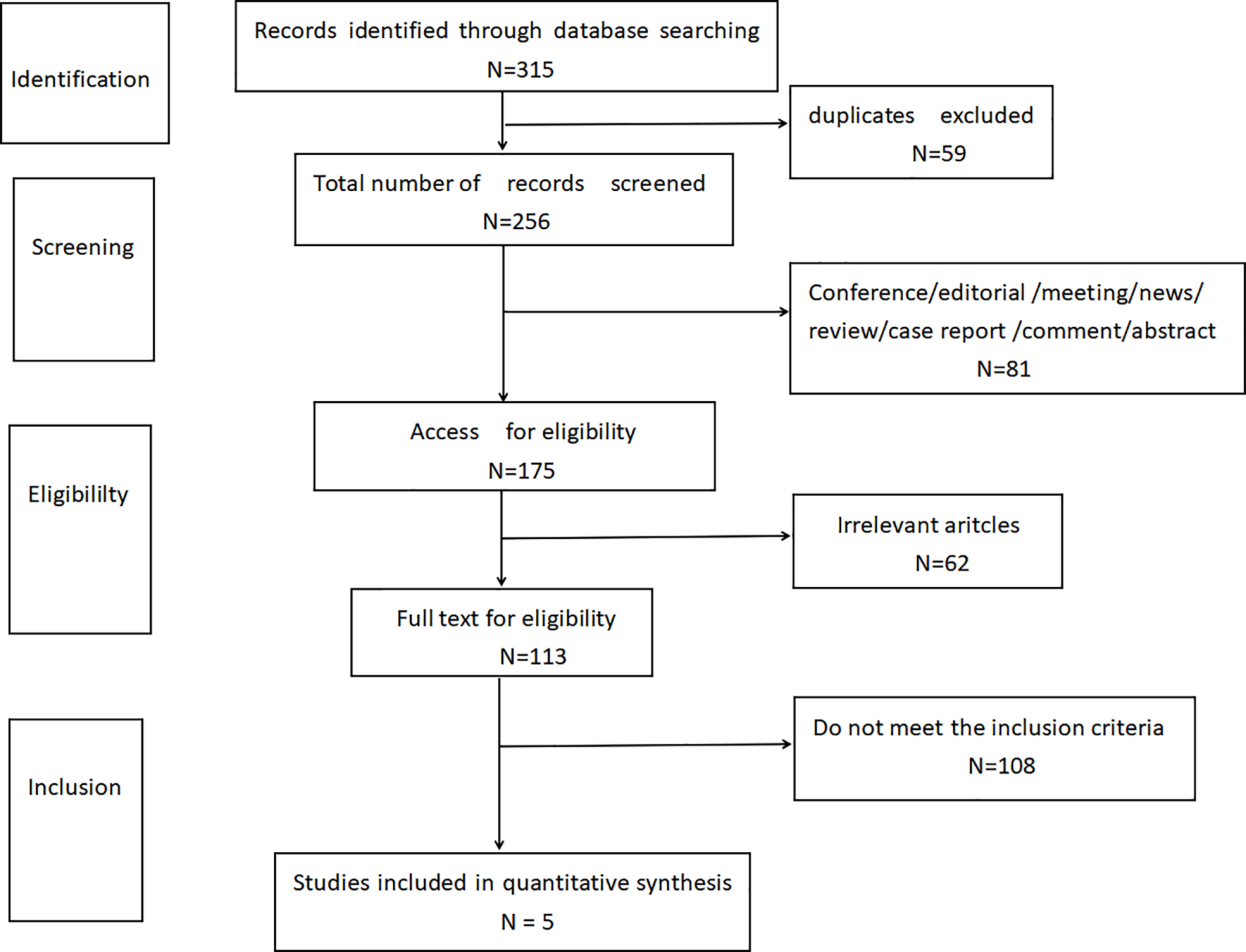
Flowchart of literature screening.

According to the limited search strategy, a total of 315 related literatures were initially detected. 59 duplicate articles were excluded, and 81 articles were excluded by reading the title and abstract, including conference, editorial, meeting, news, review, case report, comment, and abstract. By further reading the full text, 62 articles not relevant to this research topic were excluded. Finally, 108 articles were excluded according to the inclusion and exclusion criteria of this study design. Finally, according to the literature quality assessment, a total of 5 articles were included (11–15), as shown in **Figure 1**.

### Quality assessment and literature characteristics

A total of 5 studies, 174 patients, were included in this study. Study quality evaluation was performed according to MINORS. The first 8 items are for studies without control group, and each item is scored with 0 to 2 points, with a maximum score of 16 points (**Table 1**). The characteristics of the studies are shown in **Tables 2**–**5**.

**Table 1:** Quality assessment of the included studies based on the MINORS.

NO.	Author and year									Score
1	Lamiaa Zidan et al., 2021	2	2	2	2	0	2	2	1	13
2	Lisi Elizabeth Lim, et al., 2020	2	1	2	2	0	1	2	2	12
3	Amir Sabet, et al., 2017	1	2	2	2	0	2	2	2	13
4	Rahui V.Parghane, et al., 2017	2	2	2	2	0	2	2	2	14
5	Annarita Ianniello et al., 2016	2	2	2	2	0	1	2	2	13

**Table 2:** Basic characteristics of the included studies.

Author and year	Patients(n)	Age (yr) (median,range)	Histological type	Study design	Response criteria
Lamiaa Zidan et al., 2021	48 (35:13)	63 (25-84)	AC: 43	Retrospective	RECIST1.1
			TC: 5		
Lisi Elizabeth Lim, et al., 2020	48 (30:18)	68 (22-81)	AC: 32	Retrospective	Review of notes/radiology reports/correspondence
			TC: 15		
			Unknown:1		
Amir Sabet, et al., 2017	22 (16:6)	63 (42-74)	AC: 17	Retrospective	RECIST1.1
			TC: 5		
			AC: 8		
			TC: 13		
Rahui V.Parghane, et al., 2017	22 (16:6)	44 (16-72)	Small cell carcinoma: 1	Retrospective	RECIST1.1
Annarita Ianniello et al., 2016	34 (17:17)	66 (40-79)	AC: 19	Prospective	SWOG
			TC: 15		

NR, not reported; yr, year; AC, Atypical carcinoid; TC, Typical carcinoid; RECIST1.1, Response Evaluation Criteria in Solid Tumours 1.1; SWOG, Southwest Oncology Group.

**Table 3:** The treatment characteristics of the included studies.

Author and year	Dose	Cycles of therapy (median,range)	Follow-up (wk)	Cumulative activity (GBq)	DRRs (%)	DCRs (%)
Lamiaa Zidan et al., 2021	NR	4 (1-4)	6-10	27 (6-43 )	20% (10-35%)	88% (73-95%)
Lisi Elizabeth Lim, et al., 2020	4 (1-10)	1 (1-3)	NR	31.9 (7.6-49.7)	33%	83%
Amir Sabet, et al., 2017	7.8±0.6 8	4	10-14	27.2±5.9	27.3%	68.2%
Rahui V.Parghane, et al., 2017	5.55	4 (1-5)	12-16	20.1 (5.6-29.6)	31%	68%
Annarita Ianniello et al., 2016	3.7-5.5	4-5	6-8	21.5 (12.9-27.8 )	15%	62%

NR, not reported; wk, week; DRRs, disease response rates; DCRs, disease control rates.

**Table 4:** The treatment characteristics of the included studies.

Author and year	Prior therapies	Distant me tastasis	Ki67	PFS (months)(median,range)	OS (months)(median,range)
Lamiaa Zidan et al., 2021	SSA 40 (83%); Surgery 25(52%; Chemotherapy 5 (10%); Everotimus 3 (6%); Everolimus and Chemotherapy 2 (4%) Radiotherapy 2 (4%); Liver-directed therapy 2 (4%); None 1 (2%)	Local/loco-regi onal 5; Liver 10;Bone 3; Multi-organ 30	≤2%:3(6) 3o/o-20%:34 (71) >20%:3(6) UnKnown:8(17)	23 ( 18-28)	59 ( 50-)
Usi Elizabeth Lim, et al., 2020	SSA 36; PRRT6;	Liver 37; Bone 36; Lymph nodes 29; Lung 13; Subcutaneous 3 Pleura 3; Brain 3 Adrenal 2; Other{breast,gallbladd er,ovary,thyroid) 5	≤2%:15(31) 3%-20%:31(65) UnKnown:2(4)	NR	49 (3-91)
Amir Sabet, et al., 2017	Biotheraphy (16,72.7%) surgery (14, 63.6%), chemotheraphy (7,31.8%), and Iocoregional treatment (1,4.6%)	Liver 19 (86.4%); Lymph nodes10 (45.5%); bones 15 (68.2);other 6 (27.3%)	≤2%:9(41) 3%-20%:13 (59	27 (9-45)	42 (25-59)
Rahui V.Parghane, et al., 2017	Surgery 6; Chemotherapy 11;External beam radiotherapy 5; Octreotide analog 6	Liver 14; Skeleton 13; Lymph nodes 9; Lung nodules 2; Liver, skeleton and lymph nodes (widespread disease) 11	≤2%:3(13.8) 3%-20%:13(59 >20%:1(4.5) Unknown:5(22.7)	NR	40 (13-66)
Annarita Ianniello et al., 2016	No 2;Surgery 22; Somatostanin; Analogues 19; Chemotherapy 13; PRRT 9; Other 9	NR	NR	18.5(12.9-26.4)	48.6 (26.4-68.9)

NR, not reported; OS, overall survival; PFS, progression-free survival.

**Table 5:** Treatment-related toxicity of the included studies.

	Hematological toxicity n/N (%)		Nephrotoxicity n/N (%)		hepatotoxicity,n/N (%)		
Author and year	Any grade	Grade≥3	Any grade	Grade≥3	Any grade	Grade≥3	Other manifestation
Lamiaa Zidan et al., 2021	①27/48(56)	0/48(0)	NR		NR		NR
	②9/48(19)	1/48(2)					
	③12/48(25)	0/48(0)					
	④4/48(8)	0/48(0)					
	⑤24/48(50)	1/48(2)					
Lisi Elizabeth Lim, et al., 2020	16/48	2/48(AML:1MDS:1)	5/48	1/48	2/48	0/48	gastrointestinal adverse event :6;grade3 nausea:1;Superior vena cava obstruction:1
Amir Sabet, et al., 2017	2/22	2/22	0		0		0
Rahui V.Parghane, et al., 2017 Annarita Ianniello et al., 2016	NR		NR		NR		NR

① Anemia; ② leucopenia; ③Thrombocytopenia; ④ Neutropenia; ⑤ Lymphopenia; NR, not reported.

### DRRs and DCRs

**Figure 2:**
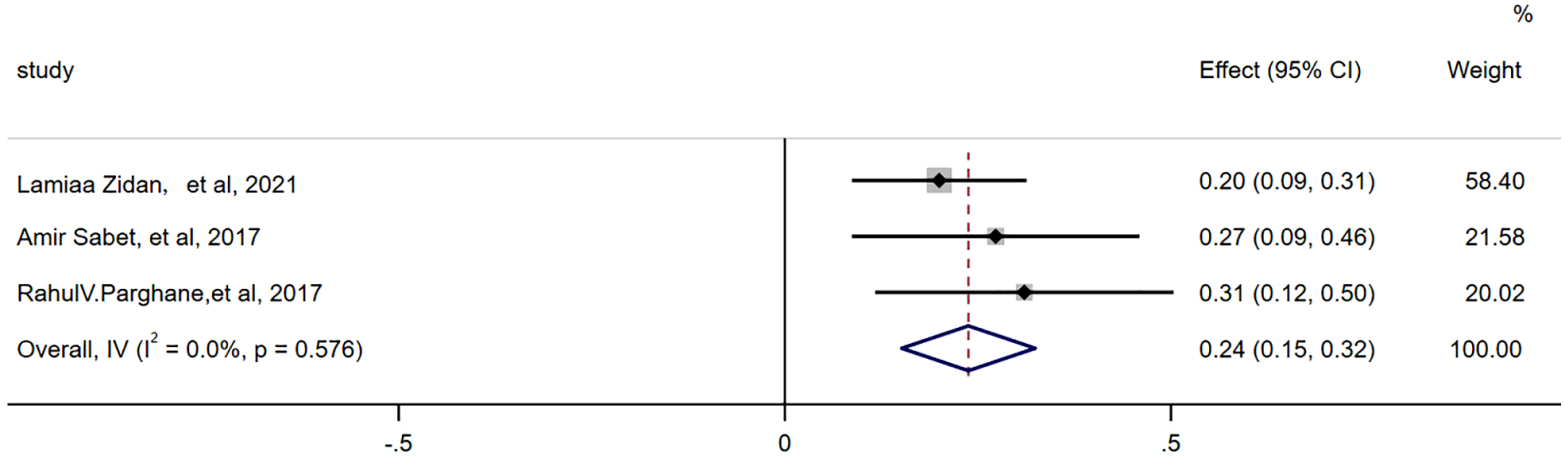
Forest plot of the proportions of disease response rates in the RECIST1.1.

**Figure 3:**
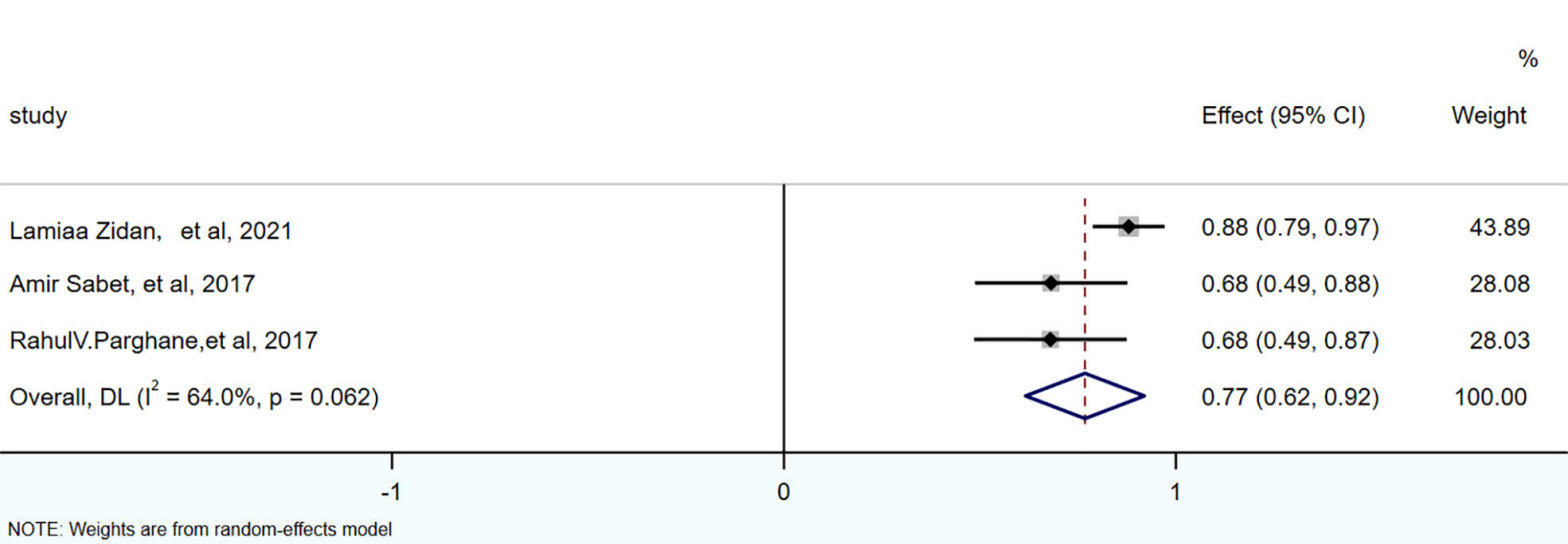
Forest plot of the proportions of disease control rates in the RECIST1.1.

There were 3 articles with a total of 92 patients using RECIST1.1 to assess the treatment response. For DRRs, the heterogeneity analysis showed that there was no significant heterogeneity (I^2 =^ 0.0%, P=0.576), so a fixed effect model was used to pool DRRs. The result of the meta-analysis showed that the pooled DRRs after treatment was 0.24 (95% CI, 0.15-0.32). For DCRs, the heterogeneity analysis showed that there was a certain degree of heterogeneity (I^2 =^ 64%, P=0.062), so a random effect model was used to pool DCRs. The results showed that the pooled rate of DCRs after treatment was 0.77 (95% CI, 0.62-0.92), as shown in **Figures 2**, **3** and in **Table 6**. There was only 1 article evaluated by SWOG criteria and 1 article was not clear, DRRs were 33% and 15%, DCRs were 83% and 62%. but they were not included in the assessment.

**Table 6:** The pools of DRRs and DCRs.

	Effects	No.of studies	Model	Pooled proportion (95% Cl)	I^2^ (%)
RECIST 1.1	Response rates	3	Fixed-effects model	0.24 (95% CI:0.15- 0.32)	0
	Control rates	3	Random-effects model	0.77 (95% CI:0.62 ~0.92)	64

### PFS and OS

**Figure 4:**
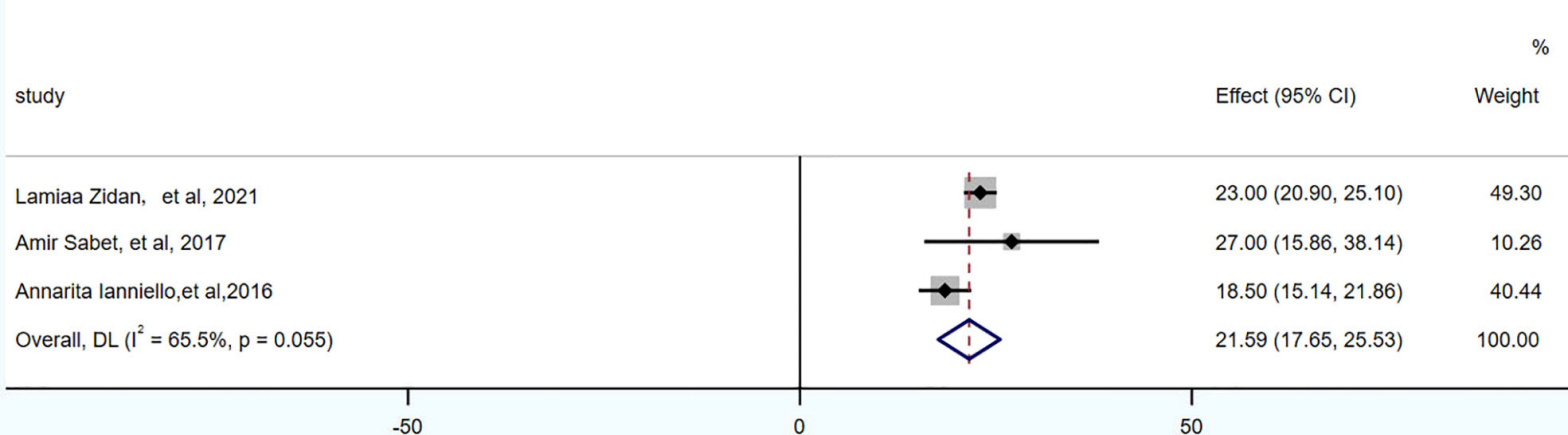
Forest plot of PFS.

**Figure 5:**
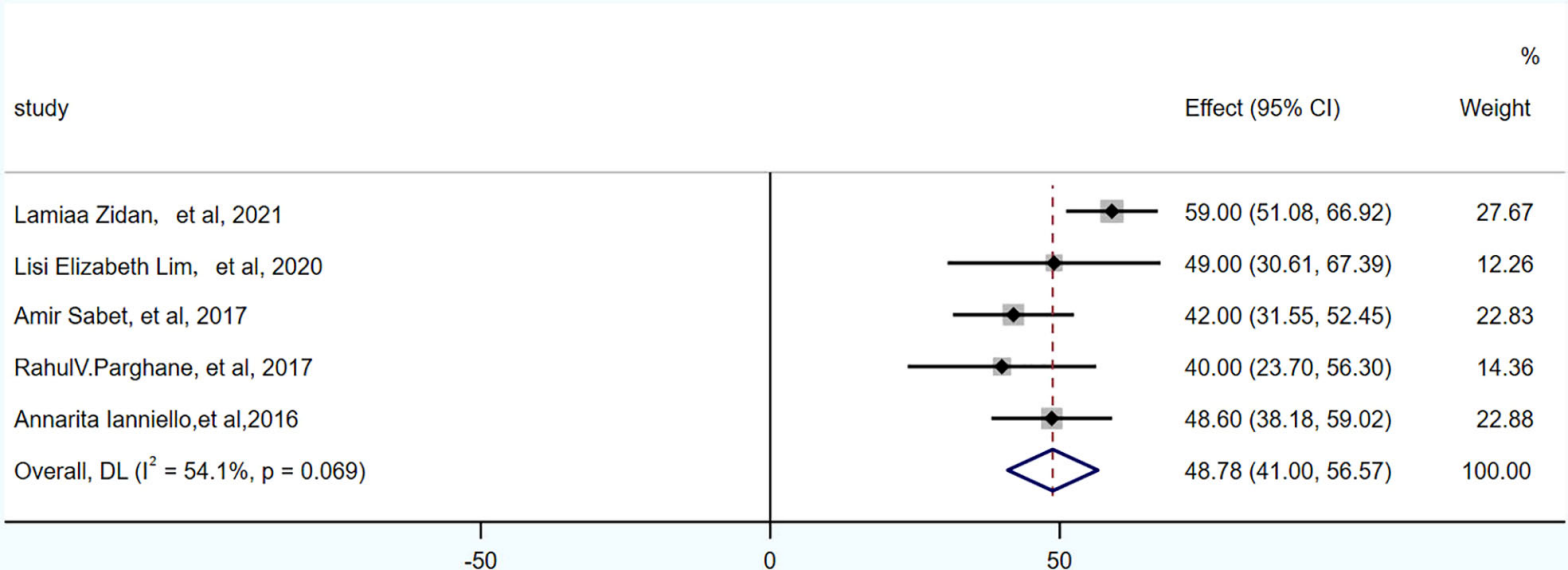
Forest plot of OS.

PFS was reported in 3 studies, 104 patients. The pooled PFS was 21.59 months (95% CI: 17.65-25.53 months). OS was reported in 5 studies, 174 patients. The pooled OS was 48.78 months (95% CI: 41-56.57 months), as shown in **Figures 4**, **5** and in **Table 7**.

**Table 7:** The pools of PFS and OS.

	No.of studies	Model	Pooled proportion (95% Cl)	I^2^ (%)
PFS	3	Random-effects model	21.59 months (95%CI:17.6 ~ 25.53 months)	65.5
OS	5	Random-effects model	48.78 months (95% Cl:41 ~ 56.57 months)	54.1

### Adverse effects

According to the CTCAE 3.0-5.0, the adverse effects of ^177^Lu-DOTATATE for pNETs were reported in all 4 studies. The most common side effects was hematological toxicity. Most patients had mild hematological toxicity, and 7 (4%) had hematological toxicity above GrIII. Among them, only 2 patients who developed AML were reported in Lisi et al (12), which resulted in the final death. One developed acute myeloid leukemia (AML) after receiving a second treatment. Another patient developed myelodysplastic syndrome (MDS) 48 months after 4 cycles of treatment, followed by fatal AML a year later. Nephrotoxicity (4%) was reported in only 7 patients, 2 studies (12, 14), and only 1 patient had nephrotoxicity above GrIII. Only in Lisi et al (12), mild hepatotoxicity was reported in 2 patients after treatment, and no hepatotoxicity above GrIII was observed. Other adverse effects included gastrointestinal adverse reactions in 6 patients (3%), nausea in 3 patients (2%), and superior vena cava obstruction in 1 patient (0.5%).

### Publication bias

**Figure 6:**
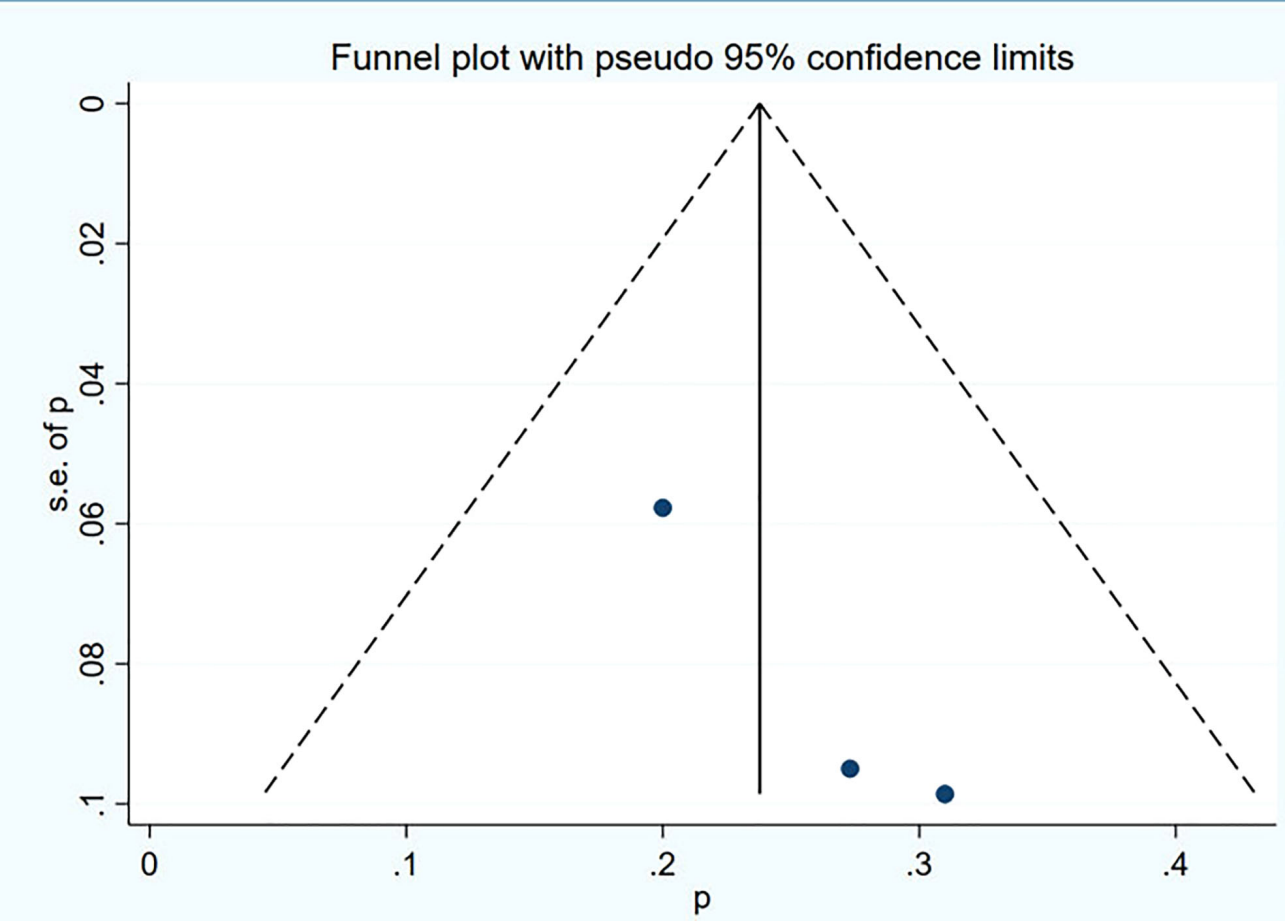
Funnel plot of disease response rates.

**Figure 7:**
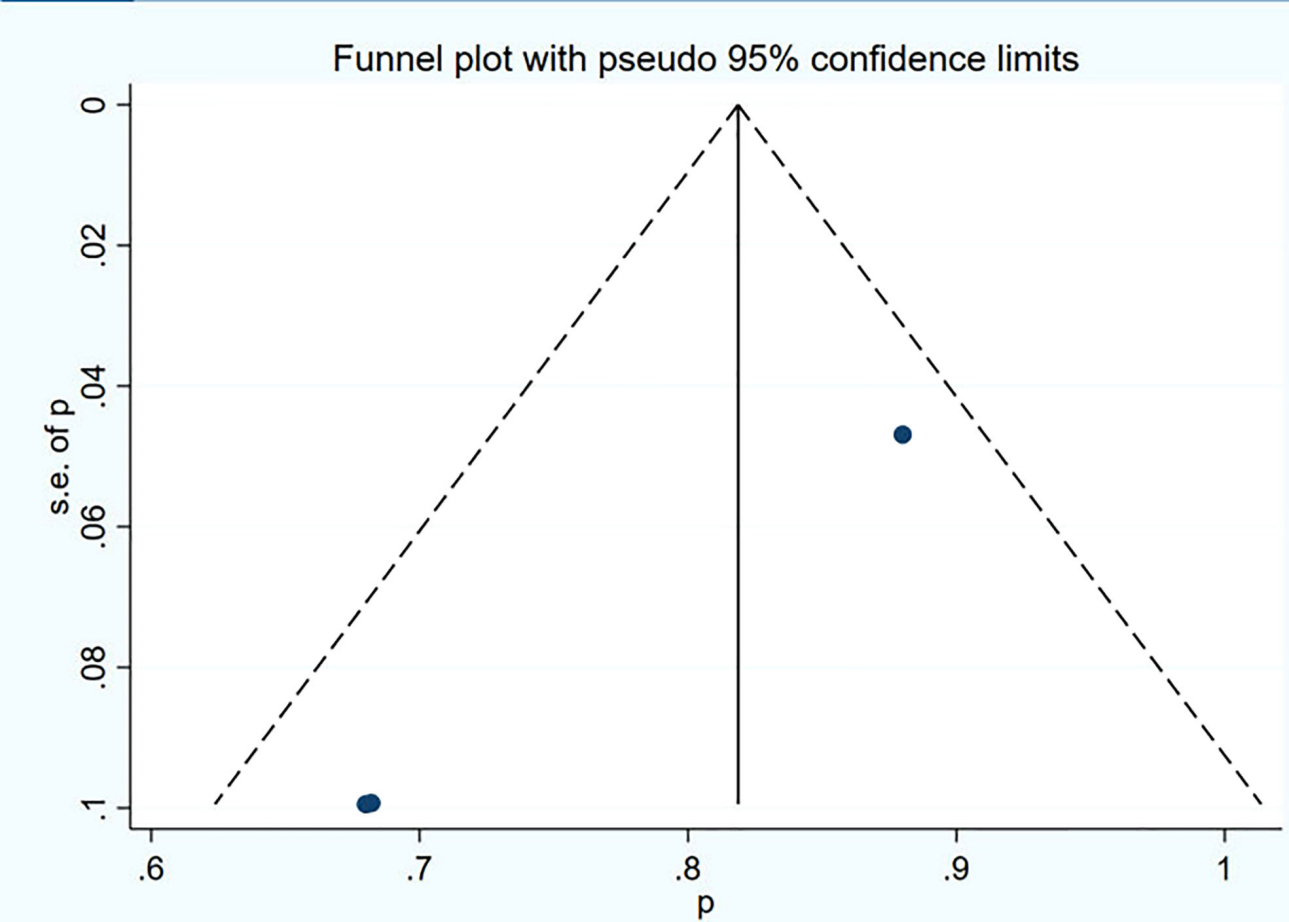
Funnel plot of disease control rates.

**Figure 8:**
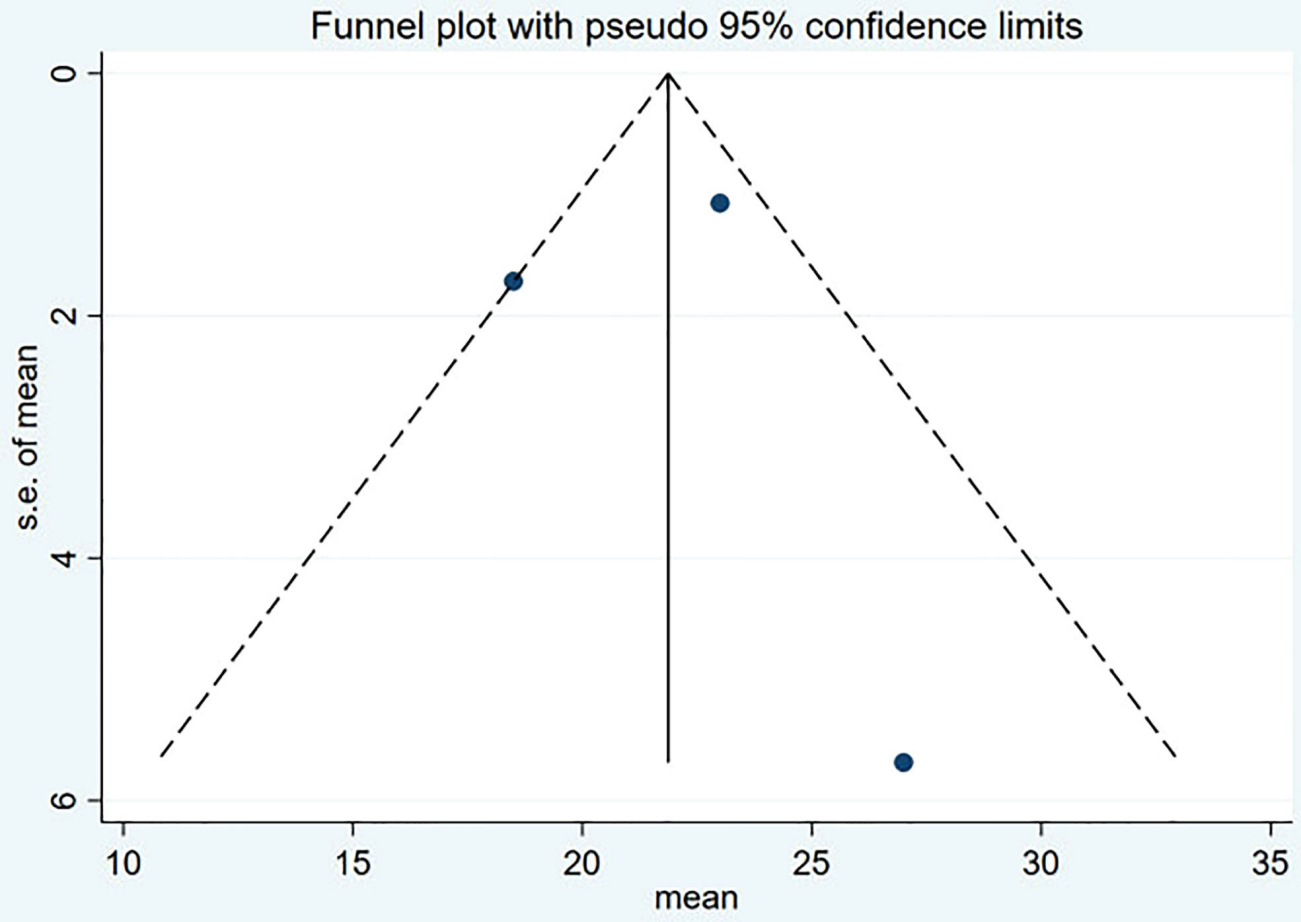
Funnel plot of PFS.

**Figure 9:**
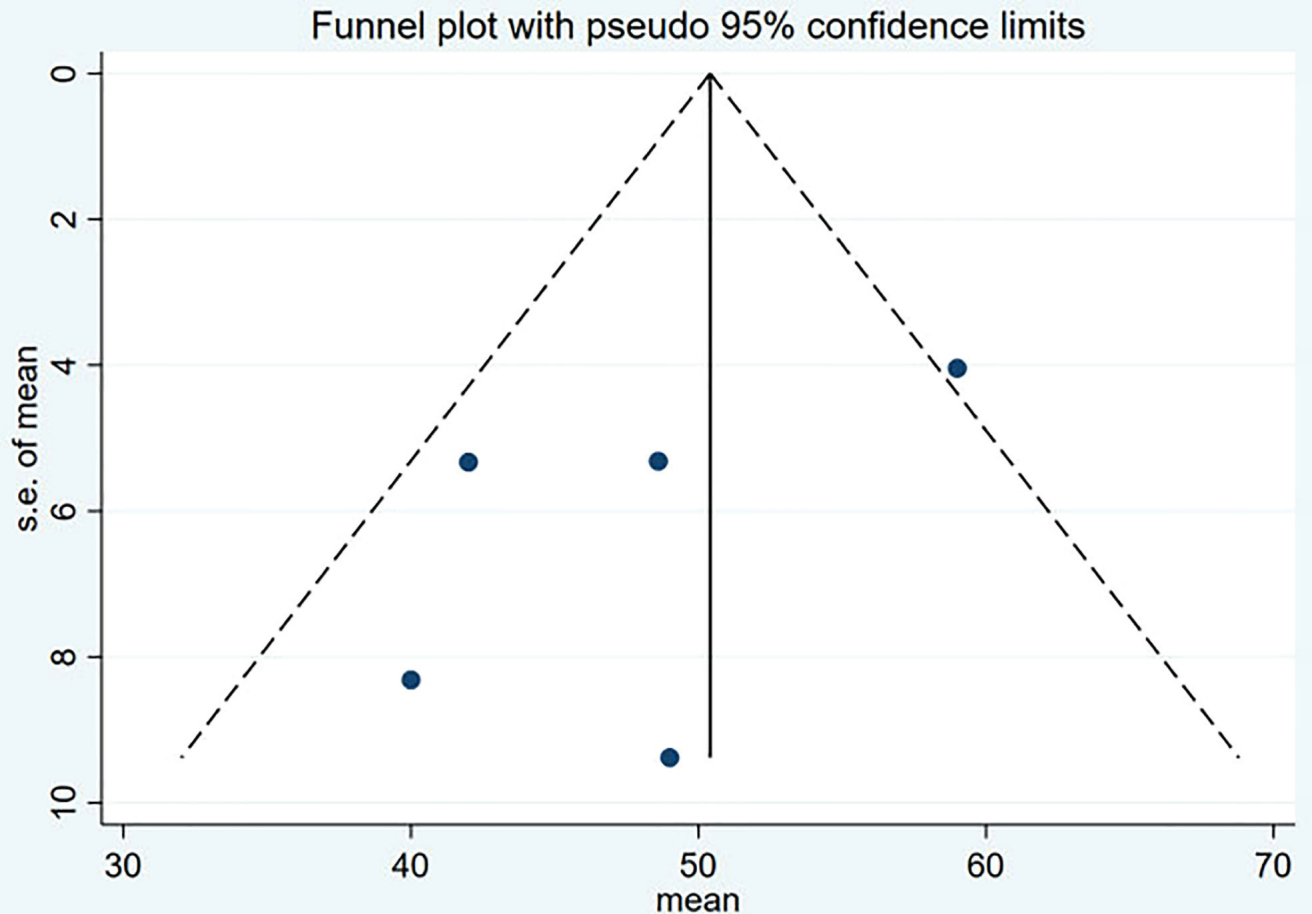
Funnel plot of OS.

Funnel plot and Egger’s test were used to assess the publication bias of the studies both qualitatively and quantitatively (**Figures 4**, **5**). The result of DRRs indicated that there was no significant publication bias (P=0.127). The result of DCRs suggested that there was a certain publication bias (P=0.003), which might be due to the fact that there were relatively few studies on ^177^Lu-DOTATATE for pNETs, and the number of studies was not enough. The results of PFS indicated there was no significant publication bias (P=0.998) and the results of OS indicated there was no significant publication bias (P=0.259), as shown in **Figures 6**–**9**.

## Discussion

Currently, studies on ^177^Lu-DOTATATE therapy for NETs are being tested in different countries. The latest NETTER-1 trial has showed that ^177^Lu-Dotatate improves OS in patients with midgut NET (7). However, there are currently only a few prospective trials of ^177^Lu-Dotatate in pNETs (16). Most data are based on mixed types of patients with primary NETs. Treatment-related data are mostly derived from small retrospective single-institutional and subgroup analyses of a few multicenter studies (17, 18). We conducted a meta-analysis of the published clinical studies of ^177^Lu-Dotatate for pNETs to evaluate its efficacy and safety. The results showed that the pooled DRRs assessed by RECIST 1.1 was 0.24 (95% CI, 0.15- 0.32) and the pooled DCRs was 0.77 (95% CI, 0.62-0.92). Similar to the previous meta-analysis of ^177^Lu-Dotatate in NETs (19, 20). The SWOG criteria was used in only one prospective study with DRRs of 15% and DCRs of 62%. Pooled OS was 48.78 months in 5 studies (95% CI: 41-56.57 months) and pooled PFS in 3 studies was 21.59 months (95% CI: 17.65-25.53 months). PRRT is often used in patients whose disease progressed after chemotherapy, SSA and other treatments. In the studies, most patients received other treatments before PRRT, and the results showed that PRRT still had good efficacy for patients whose disease progressed after these treatments. PRRT was only used as a salvage treatment in the absence of any other treatment. But one study shows, compared with chemotherapy or targeted therapy, treatment with upfront PRRT in patients with enteropancreatic neuroendocrine tumors who had experienced disease progression with SSA treatment was associated with significantly improved survival outcomes (21). Whether better outcomes can be achieved with PRRT at an early stage requires further study.

All the patients in the study had high SSTR affinity and the Krenning score were 3-4. It is unknown whether patients with Krenning score less than 3 can benefit from ^177^Lu. Besides, ^68^Ga uptake was an inclusion criterion for ^177^Lu PRRT, but it was poorly correlated with treatment response, and it was not a predictor of PRRT in patients. This confirms that SSTR expression is not the only determinant of PRRT efficacy (22). Therefore, whether patients with low SSTR expression can also benefit from ^177^Lu PRRT still needs further research and observation in the future. One study identified four parameters frequently cited as potential predictors of PRRT response: primary, PET uptake, tumor burden, and grade (ki67 index). ^18^F-FDG PET appears to play a role in predicting disease progression, tumor response, and survival in PRRT-treated patients with advanced NETs (23). Two studies (14, 15) found that patients with high FDG uptake in primary lung and/or liver metastatic lesions using dual-tracer PET-based molecular imaging for treatment response assessment had shorter OS and poorer prognosis. This suggests that patients with FDG negative baseline scans may benefit more from PRRT than positive patients. In addition, up to 50% of patients with pNETs may exhibit inter- and intra-patient heterogeneity in dual imaging with ^68^Ga-DOTATATE and ^18^F-FDG PET/CT (11). SO, although PRRT has a significant benefit in midgut NETs, there is heterogeneity in the expression of SSTR in pNETs, so further randomized trials are needed to demonstrate its effectiveness in pNETs (24, 25).

Four cases of paraneoplastic hypercalcemia associated with NETs were also reported in one study (26). One of these cases was pNET (atypical carcinoid), which may be associated with high PTHrP. Although clinical reports are rare and not found in our included patients, it represents an idea worthy of early differential diagnosis from hypercalcemia due to bone metastases. Furthermore, paraneoplastic ectopic secretion of PTHrP is often associated with poor prognosis and reduced overall survival. SSA and ^177^Lu PRRT may be more effective for long-term symptom control (27).

An interesting study investigating risk factors for gastroenteropancreatic neuroendocrine tumors found that type 2 diabetes mellitus (T2DM) and obesity were independent risk factors for GEP-NENs (28). T2DM was associated with more advanced disease and worse associated with the prognosis. These findings could have a significant impact on prevention strategies for GEP-NENs. However, whether this is also relevant in pNETs deserves attention and research.

Regarding treatment-related toxicities, the most common adverse reaction observed in all studies was hematological toxicity, which was mild in most patients, and grade 3 hematological toxicity accounted for 4.0%. In the study of Lisi et al (12), two patients developed AML, which might be due to the fact that the patients had undergone extensive chemotherapy or other treatments such as radiotherapy before PRRT. One patient developed grade 3 nephrotoxicity due to severe diarrhea caused by carcinoid syndrome, resulting in massive loss of systemic circulation. After rehydration therapy, the patient’s renal function recovered, and PRRT was continued without recurrence of nephrotoxicity. In addition, only 2 patients were observed with mild hepatotoxicity, 6 patients with gastrointestinal adverse reactions; 1 patient with grade 3 nausea and 1 patient with superior vena cava obstruction symptoms (12). In a phase 1 clinical study of ^177^Lu-DOTATATE combined with nivolumab in the treatment of pNETs, the most common adverse reaction observed was lymphopenia, and no treatment-related death occurred (17).

Our study also has some limitations and may overlook some studies not published online. The small number of studies included in this paper is the largest source of heterogeneity. Other sources may be attributable to basic characteristics of the study population, medication adherence of patients, medication dosage, etc. Due to limited data from the current study, subgroup analysis or regression analysis are not suitable to analyze the source of heterogeneity in this meta-analysis. Therefore, the results should be interpreted with caution when applied. Nonetheless, our analysis results shows good consistency compared with previous studies, indicating that ^177^Lu-DOTATATE still has a good therapeutic effect in pNETs with high SSTR expression.

## Conclusion

In conclusion, the results demonstrate that ^177^Lu-DOTATATE is effective and safe in the treatment of advanced/metastatic pNETs, delaying disease progression and improving survival. It has low side effects and high safety. However, further evaluation of its efficacy and safety in high-quality, multi-center and prospective multi-arm randomized controlled trials are needed in the future.

## Data availability statement

The original contributions presented in the study are included in the article/supplementary material. Further inquiries can be directed to the corresponding author.

## Author contributions

JM wrote this manuscript and she contributed to this work. XH, LL, and ZR collected relevant information. CZ revised the manuscript finally and provided some critical suggestions. All authors listed have read and approved this article.

## Conflict of interest

The authors declare that the research was conducted in the absence of any commercial or financial relationships that could be construed as a potential conflict of interest.

## Publisher’s note

All claims expressed in this article are solely those of the authors and do not necessarily represent those of their affiliated organizations, or those of the publisher, the editors and the reviewers. Any product that may be evaluated in this article, or claim that may be made by its manufacturer, is not guaranteed or endorsed by the publisher.
